# Action of Morphia Taken after Quinine

**Published:** 1878-04

**Authors:** 


The *Medical Brief* says: It has been noticed in several cases that when one quarter of a grain of morphine would not produce sleep, if ten grains of quinine were administered a short time previous to administering the morphine, the morphine would almost invariably act efficiently. This fact was noticed in connection with puerperal cases.—*Boston Med. and Surg. Journal.*

